# Research on Strain Measurements of Core Positions for the Chinese Space Station

**DOI:** 10.3390/s18061834

**Published:** 2018-06-05

**Authors:** Jingshi Shen, Xiaodong Zeng, Yuxiang Luo, Changqing Cao, Ting Wang

**Affiliations:** 1 School of Physics and Optoelectronic Engineering, Xidian University, Xi’an 710071, China; jingshi_shen@163.com (J.S.); xdzeng@xidian.edu.cn (X.Z.); tingw2016hg@126.com (T.W.); 2 Shandong Institute of Space Electronic Technology, Yantai 264670, China; luoyuxiang10@tsinghua.org.cn

**Keywords:** space station, strain, fiber Bragg grating sensor, transverse effect

## Abstract

The Chinese space station is designed to carry out manned spaceflight, space science research, and so on. In serious applications, it is a common operation to inject gas into the hull, which can produce strain of the bulkhead. Accurate measurement of strain for the bulkhead is one of the key tasks in evaluating the health condition of the space station. This is the first work to perform strain detection for the Chinese space station bulkhead by using optical fiber Bragg grating. In the period of measurements, the resistance strain gauge is used as the strain standard. The measurement error of the fiber optical sensor in the circumferential direction is very small, being less than 4.52 με. However, the error in the axial direction is very large with the highest value of 28.93 με. Because the measurement error of bare fiber in the axial direction is very small, the transverse effect of the substrate of the fiber optical sensor likely plays a role. The comparison of the theoretical and experimental results of the transverse effect coefficients shows that they are fairly consistent, with values of 0.0271 and 0.0287, respectively. After the transverse effect is compensated, the strain deviation in the axial detection is smaller than 2.04 με. It is of great significance to carry out real-time health assessment for the bulkhead of the space station.

## 1. Introduction

A space station is in-orbit for a long time [1] and possesses many functions not possessed by manned spacecraft, such as airlock gas recovery technology. It is essential to disembogue the gas regularly in the cabin. A space station is generally made up of a rigid metal [2], so the operation of increasing pressure will cause the deformation of the bulkhead. In order to monitor the deformation [3], a fiber Bragg grating (FBG) sensor is used to locate the specific position of the bulkhead to obtain the strain information [4]. Finally, the strain information of the space station bulkhead and the accurate assessment of health [5] are given. This is the first time that the research has been carried out on the Chinese space station, and there is no related work to which to compare it.

The FBG sensor uses optical fiber as a medium. When light enters the fiber, the reflection wavelength could be influenced by temperature, strain and other factors. By changing the wavelength, the temperature or strain information can be obtained [6,7]. It is very suitable for direct contact or indirect transmission in the process of signal monitoring [8]. The FBG sensor has the advantages of small volume, light weight, high reliability, high accuracy, anti-electromagnetic interference, long life, moisture proof and good resistance to causticity [9,10,11,12,13,14]. In particular, with the development of multiplex technology [15], FBG will have a broad application prospect.

In this paper, the main part of the cabin is pasted with FBG sensors, resistance strain gauges and the bare optical fibers. The FBG sensor with a substrate can be calibrated, and the installation is standardized. It is also not easy to damage and generate chirp. For the bare fiber, it is lightweight due to the lack of substrate. However, the installation process of the bare fiber is difficult to control, and the thickness of the adhesive layer is different. It is easy for the bare fiber to generate chirp. The substrates adopt the structure of double elastic rings. By the design, the positive and negative range can be guaranteed. The substrate is standardized, so it can be produced in enormous quantities. In the process of strain monitoring, the same process of in-orbit charging gas is also carried out. The use of resistance strain gauges is a universal method in ground testing of the spacecraft. The purpose of this work is to measure the in-orbit strain of the space station, instead of ground measurements. The resistance strain gauges cannot be used in-orbit. The main reason is that the line resistance is susceptible to the uneven distribution of temperature and bridge instability. Therefore, FBG sensors are chosen.

In this test, the FBG sensor is affixed to the bulkhead and is used to monitor the change of the bulkhead strain. This change manifests itself as a change in wavelength. Then, the demodulation equipment is used to demodulate the exact wavelength and wavelength variations, and the final bulkhead strain value is given.

The transverse effect of the sensor has a great influence on the measurement of the axial strain of the bulkhead. The axial strain is compensated by the transverse effect coefficient, and the maximum absolute error of axial strain is reduced to 2.04 με. It is of great significance for the follow-up stress analysis and safety assessment of the space station cabin.

## 2. Experiment Information

### 2.1. Principal Strain Analysis

In the structure design of the space station cabin, the maximum value of the principal strain changes in the circumferential direction, and the minimum value of the principal strain changes in the axial direction. According to the general Hooke’s law in the state of the plane stress [16], we can get:

(1) $$\varepsilon_{\max}=\frac{1}{E}\left(\sigma_{\max}-\nu \sigma_{\min}\right)$$

(2) $$\varepsilon_{\min}=\frac{1}{E}\left(\sigma_{\min}-\nu \sigma_{\max}\right)$$

where ε_max_ and ε_min_ are the maximum and minimum of the principal strain, σ_max_ and σ_min_ are the maximum and minimum of the principal stress, E is the elastic modulus and ν is Poisson’s ratio.

According to Equations (1) and (2), the principal stress can be represented as below.

(3) $$\sigma_{\max}=\frac{E}{1-\nu^{2}}\left(\varepsilon_{\max}+\nu \varepsilon_{\min}\right)$$

(4) $$\sigma_{\min}=\frac{E}{1-\nu^{2}}\left(\varepsilon_{\min}+\nu \varepsilon_{\max}\right)$$

The correct principal stress cannot be obtained unless the circumferential and axial strain is measured accurately. The first classical strength theory was adopted in damage assessment of the space station. As long as the maximum tensile stress at one point in the cabin reaches the ultimate stress of the material, brittle fracture will occur in the material, which may cause a disaster on the space station.

### 2.2. Research Methodology

**Figure 1:**
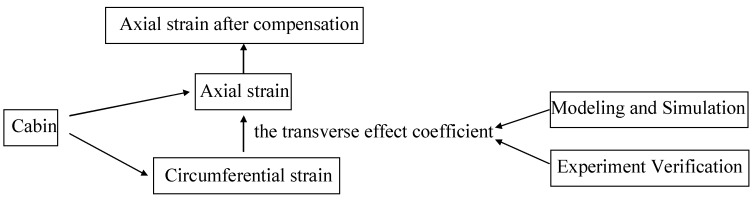
A graph with the mentioned methodology.

Figure 1 is a flow-process diagram for the mentioned methodology. We measured the structural deformation of the space station under different conditions in this paper. The use of resistance strain gauges is a common method in ground testing of the spacecraft. FBG sensors were used to perform in-orbit testing in this work. Therefore, the results of the two measurement methods need to be compared. The materials of the cabin and cantilever beam were completely identical. In the test conditions, the mechanical conditions of the bulkhead were similar to that of the cantilever beam, and the use of the cantilever beam model was rather helpful for performing an accurate and quantitative analysis.

### 2.3. Devices

**Figure 2:**
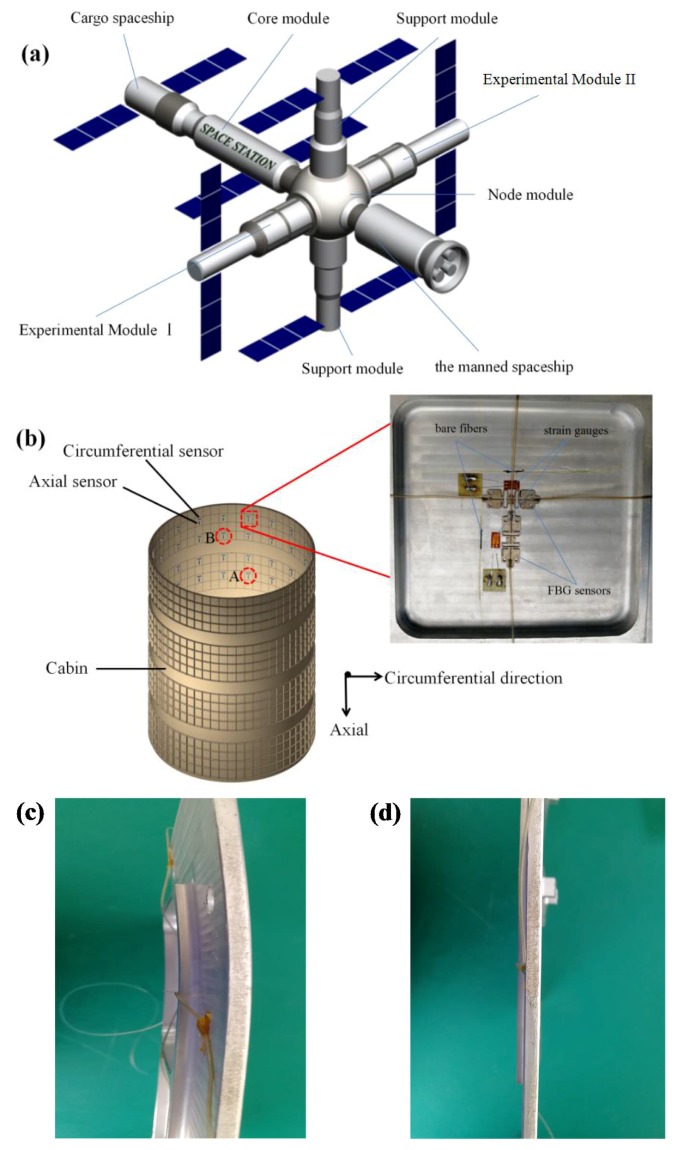
Schematic of the space station (**a**) and installation of FBG sensors in the cabin (**b**). Top view of the bulkhead (**c**) and right view of the bulkhead (**d**).

The space station consists of the core module, the node module, the module I, the experimental module II, the cargo spaceship, the manned spaceship, and so on, as shown in Figure 2a. For the strain monitoring of the space station, FBG sensors with the substrate were attached to the important area in the circumference and the axial direction, as shown in Figure 2b. In this area, the reinforced structure was specially designed, and the front view is displayed in the upper right corner of Figure 2b.

The substrate is a necessary condition to avoid fiber damage. The substrate of the FBG sensors is a 304 stainless steel substrate. FBG sensors are fixed to the bulkhead by gluing, and the model of the adhesive is J133. In this paper, the length of the substrate was 36 mm, the width 12 mm and the thickness 1.3 mm. The interrogator used to measure the wavelength was the Micron Optics Int. (MOI) interrogator “si155” from the United States. For the FBG sensing demodulation, wavelength division multiplexing technology was adopted with a spectrum range of 1525–1565 nm. The center wavelengths were 1529.9 nm, 1534.4 nm, 1538.9 nm, 1543.9 nm, 1547.2 nm, 1552.2 nm, 1556.7 nm and 1561.2 nm. The fiber had good radiation resistance. The space station has adopted the irradiation-resistant optical fiber Nufern, and it is carved by the phase mask method. Here, the change of ambient temperature has a great impact on the accuracy of the corresponding measurement. FBG sensors are used as temperature sensors. The method used in the test was to paste a temperature sensor on the space station bulkhead. The FBG temperature sensor adopts a sandwich structure. Since the temperature sensor was placed in a floating position, the temperature measurement value was not affected by the strain on the cabin. It is only affected by temperature changes and can therefore be used for temperature compensation for strain measurements.

The resistance strain gauges are used in the same direction near each FBG sensor. The details of the resistance strain gauge we used are as follows: (1) dimensions: 6.4 × 3.5 mm; (2) 3 cm silver-plated copper wire; (3) base material: phenolic resin; (4) manufacturer: Zhonghang Electronic Measuring Instruments Co. Ltd. in the city of Xi’an, China; (5) sensitivity coefficient: 2.22. The distance between the resistance strain gauge and the FBG sensor was around 2 mm. The gas was injected into the tank. When the pressure was stable, the measured values of the FBG sensor and the resistance strain gauge were recorded. The process of increasing pressure was performed 10 times, starting from 50 kPa and increasing by 5 kPa each time.

Figure 2b is a schematic view of FBG sensors stuck to a columnar bulkhead. The size of the square in the front view is 10 × 10 cm. The specific paste position is shown in the front view. The top view and right view are shown in Figure 2c,d. There are eight FBG sensors on each fiber, using wavelength division multiplexing. A total of eight fiber channels was used for the parallel detection mode. Therefore, there was a total of 64 FBG sensors. There were two FBG sensors at each measurement point. There was a total of 32 measurement points, and the adjacent monitoring points were separated by about 20 cm. The reason for the choice is that the core position of the cabin is vulnerable to damage. Figure 2c is the top view of the bulkhead, and Figure 2d is the right view of the bulkhead. The front view is a test figure for actual measurements, which includes FBG sensors, bare fibers and strain gauges, as shown in Figure 2b.

## 3. Results Analysis

### 3.1. Strain Measurement in the Axial and Circumferential Direction

The FBG sensor has an original center wavelength, which is reflected by the FBG sensor changes, when the external strain changes. The reflection wavelength value of the FBG at each moment can be demodulated to obtain the wavelength variation. Finally, the strain value to be measured is obtained through the sensitivity coefficient.

**Figure 3:**
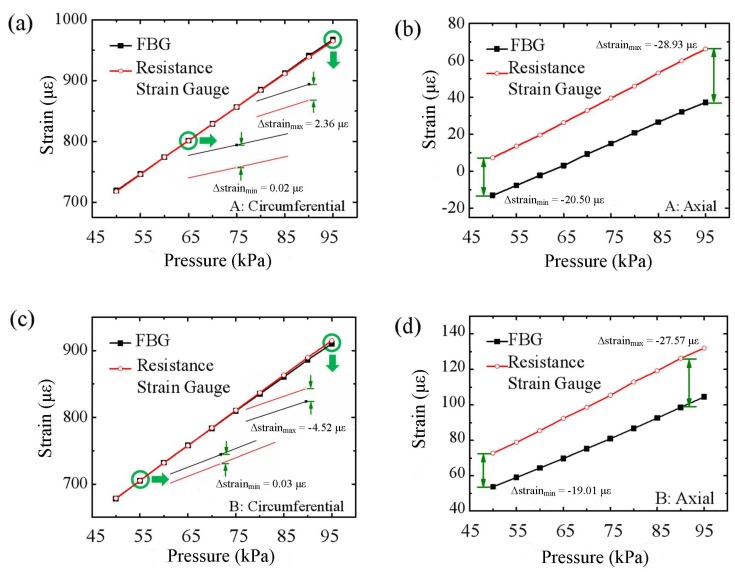
The strain values of Points A and B in the axial and circumferential direction at different pressures. (**a**) Point A: circumferential strain measurements; (**b**) Point A: axial strain measurements; (**c**) Point B: circumferential strain measurements; (**d**) Point B: axial strain measurements.

In the process of increasing the gas pressure in the cabin, 10 typical strain measurements are recorded. As an example of Points A and B in Figure 1b, the strain of the space station cabin is detected in the axial and circumferential directions, as shown in Figure 3.

The results of the real-time measurement of the experiment are shown in Figure 3. Figure 3a,c shows the results of circumferential strain measurements at Points A and B in the pressurization process separately. As shown in Figure 3b,d, the measurement results of the axial strain on Points A and B are given.

The pressure sensor used for calibration is a pressure level transmitter. As the pressure gradually increases, the deformation of the bulkhead also gradually becomes larger. That is, the pressurization process results in the prerequisite of an increase in the deformation. Figure 3 shows the measured strain values of both FBG sensors and resistance strain gauges at each pressure value.

From Figure 3, it can be seen that under two sensors, the results of the circumferential strain measurements of A and B are very close, but the results of the axial strain are very different. Table 1 shows the difference between the measured strain of FBG sensors and that of the resistance strain gauges at each pressure value in Figure 3, that is the absolute error. Compared with the absolute error of strain, it can be concluded that the FBG sensors have large errors in measuring axial strain and small errors in measuring circumferential strain. However, the measurement error is very small when comparing the strain of the bare fiber and the resistance strain gauge in the axial direction.

FBG sensors have metal substrates. Bare fibers have no substrate. The axial strain measurement errors of Points A and B of the FBG sensors are one order of magnitude larger than that of bare fiber. Therefore, the transverse effect of the FBG sensor with the substrate may lead to strain measurement error. It is necessary to calculate the transverse effect coefficient and complete the axial strain compensation.

### 3.2. Modeling and Simulation

**Figure 4:**
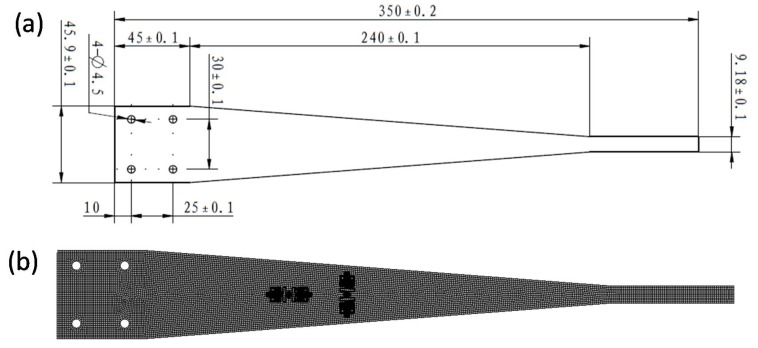
The simulation model of the sensor and the cantilever beam: (**a**) modeling dimensions of the cantilever beam; (**b**) the position of the FBG sensors on the cantilever beam.

The size of the cantilever beam model is shown in Figure 4a. On the cantilever beam, the FBG sensor model with the substrate is set along the length and width of the beam, as shown in Figure 4b. 

$\varepsilon_{v}$ is defined as the width direction strain of the beam surface, ε as the strain in the length direction of the beam surface, $\varepsilon_{fv}$ as the strain of the FBG sensor in the width direction of the beam and $\varepsilon_{f}$ as the FBG sensor strain on the length direction of the beam.

The transverse effect coefficient α is expressed as:

(5) $$\alpha =\frac{\varepsilon_{fv}}{\varepsilon_{f}}-\frac{\varepsilon_{v}}{\varepsilon }$$

The definition of ratio coefficient “*η*” of strain in the length direction between the FBG and the beam is $\eta =\frac{\varepsilon_{f}}{\varepsilon }$. The ratio coefficient “*η_v_*” of strain between the FBG and the beam in the width direction is defined as $\eta_{v}=\frac{\varepsilon_{fv}}{\varepsilon_{v}}$.

According to the simulation, we can get η = 1.0211, $\eta_{v}$ = 1.1069.

The strain ratio of the beam along the width and length direction obtained through the simulation is 0.322. That is:

$$\frac{\varepsilon_{v}}{\varepsilon }=0.322$$

Take the value of *η*, *η_v_* and *ε_v_*/*ε* into Equation (1). From this, we can get that the transverse effect coefficient of 0.0271.

### 3.3. Experiment Verification

FBG sensors and resistance strain gauges are connected to the length and width of the cantilever beam, respectively. Here, the strain in the length direction of the beam (in the long axis direction) in the resistance strain gauge is defined as *ε*_1_. The strain on the width of the beam (in the short axis direction) is defined as *ε*_2_. The strain in the width direction of the resistance strain gauge beam (in the short axis direction) is defined as *ε*_3_. The cantilever beam exerts its weight at a certain time. The results of the test are shown in Table 2.

The test data in Table 2 were obtained through a cantilever test. The 18 conditions correspond to the process of increasing from 0–17 weights in order. Each time different weights are added, strain measurements of both FBG sensors and resistance strain gauges are performed.

The fitting curves of *ε*_1_–*ε*_3_ and *ε*_2_–*ε*_3_ are almost linear. The slope is −0.3693 and −0.3406, respectively. The difference in slope comes from the transverse effect coefficient, which is 0.0287. The simulation results of the above-mentioned transverse effect coefficient are in good agreement with the experimental results of the axial strain compensation coefficient of the FBG sensor and the substrate.

### 3.4. Relationship between Strain Based on the FBG Sensor and the Strain of the Cylindrical Cabin

**Figure 5:**
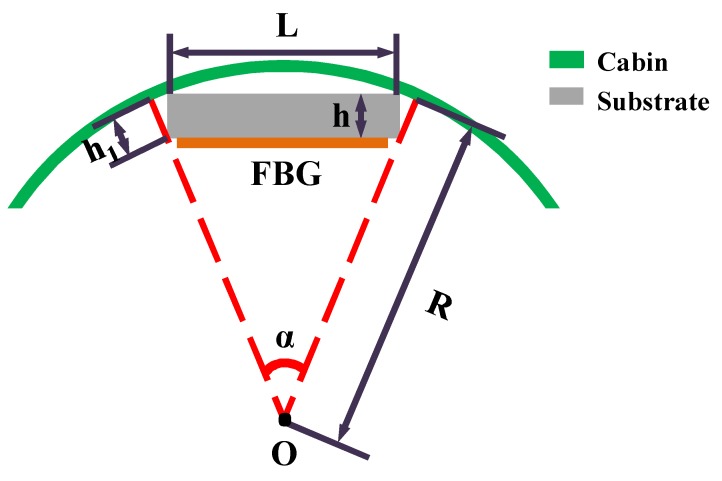
The top view of cabin pasted with the FBG sensor.

Because the cross-section of the cabin is round, as shown in Figure 5, when increasing the air pressure, we need to consider the relationship between the strain measurement of the FBG sensor and the actual strain of the cylinder cabin.

During the process of filling the gas, the change of the internal pressure directly leads to the change of the strain in the cabin, and the bulkhead moves to the radial direction. The length of the sensor is defined as *L* before the cabin moves forward, and the radius of the cylinder cabin is defined as *R*. After the cabin is moved, the length of the sensor is defined as *L′*, and the cabin radius is defined as *R′*. The strain measurement of FBG is defined as *ε_f_*, the height of the substrate of FBG sensor as *h* and the angle *α* as the central angle of the circle, as shown in Figure 5.

Before and after the cabin is moved, the length of the sensor can be expressed as:

(6) $$\frac{L}{2}=(R-h_{1})\cdot \sin\frac{\alpha }{2}$$

(7) $$\frac{L^{\prime }}{2}=(R^{\prime }-h_{1})\cdot \sin\frac{\alpha }{2}$$

According to Equations (6) and (7), it can be obtained:

(8) $$\frac{\Delta L}{2}=\Delta R\cdot \sin\frac{\alpha }{2}$$

Then, we can know:

(9) $$\Delta L=\frac{\Delta R\cdot L}{R-h_{1}}$$

For the FBG sensor,

(10) $$\Delta L=\varepsilon_{f}\cdot L$$

Substituting Equation (10) into Equation (9), it has:

(11) $$\Delta R=\varepsilon_{f}\cdot (R-h_{1})$$

The cabin strain can be defined as:

(12) $$\varepsilon =\frac{\Delta R}{R}$$

According to Equations (11) and (12), the strain of the cabin can be written as:

(13) $$\varepsilon =\frac{\varepsilon_{f}\cdot (R-h_{1})}{R}$$

Here,

(14) $$h_{1}\approx h\cdot \cos\frac{\alpha }{2}$$

Equation (14) is substituted into Equation (13), then it has:

(15) $$\varepsilon =\frac{\varepsilon_{f}\cdot (R-h\cdot \cos\frac{\alpha }{2})}{R}$$

Considering the actual size, we can get:

(16) $$R-h\cdot \cos\frac{\alpha }{2}\approx R$$

On the basis of Equation (15) and Equation (16), we can get:

(17) $$\varepsilon \approx \varepsilon_{f}$$

Therefore, the strain measurement of the FBG sensor with the substrate is approximately equivalent to the actual strain of the cylindrical cabin.

### 3.5. Compensation of Axial Strain

**Figure 6:**
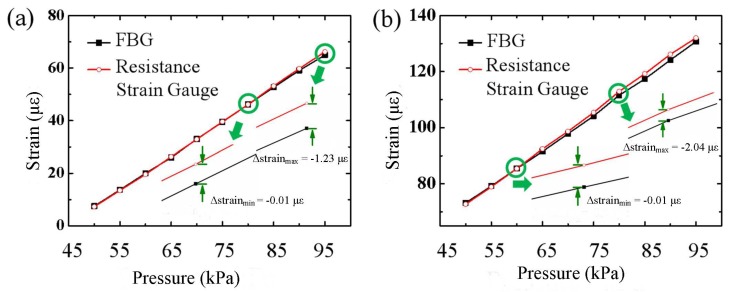
The comparison of the measurement results of the FBG after compensation and resistance strain gauge for Point A (**a**) and Point B (**b**).

Considering the influence of the transverse effect, we need to compensate the axial strain measurement of FBG sensors, as shown in Figure 6. 

Figure 6 shows the measurement results at Points A and B between the FBG sensor after compensation and the resistance strain gauge. Before compensation, for the axial strain, the strain measurement error of the FBG sensor was large, and the maximum absolute error was 28.93 με. After compensation, the strain of the FBG sensor was quite consistent with the strain of the strain gauge. The maximum absolute error of the axial strain was 2.04 με. The results show that the transverse effect of the substrate is the main source of the measurement error of the axial strain of the FBG.

## 4. Conclusions

The space station has a long working cycle and needs to be filled with gas for daily life and scientific experiments. The FBG sensor was selected for the first time in the Chinese space station to monitor the strain of the cabin. The resistance strain gauge is used as the strain measurement standard. The results show that the strain measurement of the FBG sensor and the resistance strain gauge are very close in the circumferential direction. However, the absolute error is quite large in the axial direction. Adopting the axial compensation of the transverse effect coefficient, the absolute error is less than 3 με. These results are of great help to the test of force and the accurate assessment of the state in the cabin.

## Figures and Tables

**Table 1:** The absolute error of strain for Points A and B in the circumferential and axial direction.

	Point	A (με)		B (με)		Bare Fiber (με)	
Pressure Values		Circumferential	Axial	Circumferential	Axial	Circumferential	Axial
50		1.26	−20.50	0.10	−19.01	0.14	−0.20
55		0.30	−21.20	0.03	−19.93	0.26	−0.13
60		0.04	−21.88	0.46	−21.02	0.22	0.52
65		0.02	−23.29	0.21	−22.65	0.53	0.98
70		0.18	−23.66	−0.40	−23.36	−0.17	1.14
75		0.17	−24.67	−0.75	−24.54	1.24	1.25
80		0.40	−25.39	−1.86	−26.25	0.59	1.44
85		1.07	−26.63	−2.85	−26.61	0.94	1.82
90		1.98	−27.72	−3.44	−27.57	2.11	2.25
95		2.36	−28.93	−4.52	−27.53	2.45	2.14

**Table 2:** The strain measurement results of FBG sensors and resistance strain gauges.

*ε*_1_/με	*ε*_2_/με	*ε*_3_/με
0.0	0.0	0.0
−26.8	−30.7	78.6
−53.3	−59.1	157.7
−80.1	−88.7	236.6
−106.9	−120.5	316.1
−133.4	−151.2	395.6
−160.4	−181.9	474.5
−187.3	−210.3	552.7
−214.4	−241.0	631.7
−241.3	−269.4	710.5
−268.3	−299.0	789.2
−295.2	−326.3	868.5
−322.1	−355.8	947.0
−349.2	−382.0	1026.3
−375.9	−410.4	1105.3
−402.9	−438.8	1184.5
−429.7	−466.1	1263.6
−457.0	−496.8	1342.6
